# Association of Maternal Dietary Habits and *MTHFD1* Gene Polymorphisms With Ventricular Septal Defects in Offspring: A Case-Control Study

**DOI:** 10.3389/fped.2021.785440

**Published:** 2022-02-02

**Authors:** Xinli Song, Yiping Liu, Tingting Wang, Senmao Zhang, Mengting Sun, Jing Shu, Jianhui Wei, Jingyi Diao, Jinqi Li, Yihuan Li, Letao Chen, Ping Zhu, Jiabi Qin

**Affiliations:** ^1^Department of Epidemiology and Health Statistics, Xiangya School of Public Health, Central South University, Changsha, China; ^2^National Health Committee Key Laboratory of Birth Defect for Research and Prevention, Hunan Provincial Maternal and Child Health Care Hospital, Changsha, China; ^3^Guangdong Academy of Medical Sciences, Guangdong Cardiovascular Institute, Guangdong Provincial People's Hospital, Guangzhou, China; ^4^Hunan Provincial Key Laboratory of Clinical Epidemiology, Changsha, China

**Keywords:** ventricular septal defects, *MTHFD1* gene, interaction effects, case-control study, dietary habits

## Abstract

**Objectives:**

This study aimed at assessing the association between maternal methylenetetrahydrofolate dehydrogenase 1 (*MTHFD1*) gene polymorphisms, maternal dietary habits, and their interactions with the risk of ventricular septal defects (VSD) in offspring.

**Methods:**

From November 2017 to March 2019, a case-control study comprising 360 mothers of VSD cases and 504 mothers of healthy infants was conducted in Han Chinese populations. The main exposures of interest were maternal dietary habits in early pregnancy and *MTHFD1* gene polymorphisms. Logistic regression models were used to estimate the main effects and interaction effects.

**Results:**

It was observed that maternal excessive intake of pickled vegetables (aOR = 1.85, 95%CI: 1.45–2.37), smoked foods (aOR = 1.93, 95%CI: 1.48–2.51), barbecued foods (aOR = 1.74, 95%CI: 1.28–2.36), and fried foods (aOR = 1.68, 95%CI: 1.30–2.17) were associated with a higher risk of VSD in offspring, whereas maternal excessive intake of fresh meat (aOR = 0.61, 95%CI: 0.47–0.79), fish and shrimp (aOR = 0.29, 95%CI: 0.23–0.38), fresh eggs (aOR = 0.54, 95%CI: 0.42–0.70), fresh fruits or vegetables (aOR = 0.44, 95%CI: 0.33–0.60), soy foods (aOR = 0.65, 95%CI: 0.53–0.80), and milk products (aOR = 0.49, 95%CI: 0.40–0.59) could contribute significantly to a lower risk of VSD in offspring. Furthermore, the genetic polymorphisms of maternal *MTHFD1* gene at rs1950902 (GA vs. GG: aOR = 0.67, 95%CI: 0.50–0.90) and rs2236222 (GG vs. AA: aOR = 2.75, 95%CI: 1.57–4.83) were significantly associated with the risk of VSD in offspring. In addition, there was a significant interaction effect between maternal dietary habits and *MTHFD1* gene polymorphisms on the risk of VSD.

**Conclusions:**

Maternal dietary factors, *MTHFD1* genetic polymorphisms, and their interactions were all associated with the risk of VSD in offspring. However, further research in diverse ethnic populations and with a larger sample size is warranted to corroborate our findings.

**Trial Registration:**

Registered in Chinese Clinical Trial Registry Center; registration number, ChiCTR1800016635; registration date, 06/14/2018 (Retrospectively registered); URL of trial registry record, https://www.chictr.org.cn/showproj.aspx?proj=28300.

## Introduction

Congenital heart disease (CHD) refers to a group of anatomic heart and great vessel malformations that arise during the embryologic development of the fetus. CHD is one of the most prevalent birth defects, affecting around 2.50 out of every 1,000 births in China (1), and it imposes a substantial burden on society and families, making it a serious public health issue. Ventricular septal defects (VSD) are the most common congenital cardiac malformations (2). It is generally understood that folic acid supplementation is the most effective large-scale strategy to date for decreasing CHD, particularly for conotruncal defects (CTD) and VSD (3, 4). Also, it has been proven that CTD and VSD likely share some risk factors (5); both are likely associated with folate metabolism (6). However, the underlying etiology of VSD, which comprises a simple or complicated combination of genetic, environmental, and lifestyle factors, is not fully known.

Early work in our laboratory and other literature reveal that genetic polymorphisms of genes related to the maternal folate-homocysteine metabolism pathway, such as methylene-tetrahydrofolate reductase (MTHFR) (7), cystathionine beta synthase (CBS) (8), and methionine synthase (MS) (9), are closely associated with the development of CHD. In this study, the gene of interest is methylenetetrahydrofolate dehydrogenase 1 (*MTHFD1*), which has gotten less attention than the abovementioned genes despite its importance in one-carbon folate metabolism. The *MTHFD1* gene encodes a trifunctional enzyme that includes 5,10-methylenetetrahydrofolate dehydrogenase, 5,10-methenyltetrahydrofolate cyclohydrolase, and 10-formyltetrahydrofolate synthetase (10). MTHFD1 catalyzes three sequential reactions that interconvert tetrahydrofolate (THF) to 5,10-methylenetetrahydrofolate (5,10-methylene THF) (10, 11). Of note, 5,10-methylene THF has a wide range of applications, including purine, thymidylate, and serine synthesis as well as regenerating methionine from homocysteine in the methylation cycle (12, 13). Because of the significance of the MTHFD1 enzyme, it is assumed that abnormal expression or decreased activity could cause a folate-homocysteine imbalance and interfere with DNA synthesis. Experiments indicate that the *MTHFD1* gene with mutant genotypes expresses less stable *in vitro* and less active *in vivo* MTHFD1 protein, disrupting *de novo* purine and DNA synthesis (14, 15). Furthermore, previous epidemiologic studies show that functional non-synonymous single nucleotide polymorphism (SNPs) of the *MTHFD1* gene, such as *MTHFD1* rs2236225 (G1958A; R653Q) and *MTHFD1* rs1950902 (C401T; R134K), are associated with an increased risk of CHD, albeit the associations are controversial (14, 16, 17). To provide more epidemiological data, this study focuses on VSD, including conotruncal VSD (CTD cases with VSD), and investigates the association between maternal SNPs of the *MTHFD1* gene and the risk of VSD in Chinese populations.

Dietary habits, as a crucial modifiable influencing factor, are shown to influence pregnancy outcomes, including birth defects. To date, the majority of attention has been focused on the role of nutrients or food items alone or in conjunction with dietary patterns in the development of CHD (18–20). However, given the diversity in socioeconomic status, races, geographic location, and culture, it is apparent that dietary factors vary by population. As a result, this study focuses on the associations between maternal dietary habits and the risk of VSD in offspring, which is helpful for accurate CHD prevention. In addition, previous study has discovered a substantial relationship between dietary habits and serum and red blood cell folate levels (21, 22). According to Christensen's study, low dietary folate interacts with MTHFD1 synthetase deficiency in mice, a model for the R653Q mutation, to significantly increase the risk of birth defects (22). Thus, we hypothesize that the maternal *MTHFD1* gene and dietary habits combine to increase the risk of VSD. Based on the abovementioned background, we performed a hospital-based, case-control study in Han Chinese populations to (1) assess the association between maternal dietary habits in the first-trimester pregnancy and the risk of VSD, (2) determine the relationship between the maternal *MTHFD1* gene polymorphisms and the risk of VSD, and 3) investigate the interaction effect of maternal *MTHFD1* gene polymorphisms and dietary habits on the risk of VSD.

## Materials and Methods

### Recruitment of Study Participants

The recruitment was conducted by the Hunan Provincial Children's Hospital (Changsha, Hunan Province, China) from November 2017 to December 2019. A case-control design was adopted in the present study. Mothers of infants with VSD were assigned to the case group, whereas mothers of infants who did not have a congenital malformation following medical examination were assigned to the control group. Participants in the research were recruited from two distinct clinics within the same hospital. VSD children were consecutively recruited from the Department of Cardiothoracic Surgery, and the control children were randomly recruited from the Department of Child Healthcare after health counseling or medical examination during the same study time as the cases. It is worth noting that the recruiting location we chose is well-known in the province because of its outstanding CHD diagnosis and treatment capabilities. As a result, this hospital's CHD patient source covers the whole province, indicating a good representation of cases. We ensured that all children in the case and control groups were < one 1 year old to decrease maternal recall bias for exposures prior to the current pregnancy and in early pregnancy. We only included individuals of Han Chinese descent because a homogeneous ethnic background may eliminate residual confounding effects from genetic and cultural differences. Thus, all eligible participants should fulfill the inclusion criteria as follows: (1) provided informed consent, (2) completed the questionnaire, (3) singleton pregnancy, (4) spontaneous pregnancy, (5) Han Chinese population, and 6) provided the blood sample. Of note, this study only included non-syndromic VSD; those with syndromic VSD, such as those with additional organ malformations or known abnormalities, were excluded.

Our study was approved by the ethics committee of the Xiangya School of Public Health of Central South University, and written informed consent was obtained from all mothers. Besides this, we have registered this study in the Chinese Clinical Trial Registry Center (registration number: ChiCTR1800016635).

### Information Collection

The outcome of interest in this study was VSD, which was defined using the Chinese Surveillance of Birth Defects Classification System. All VSD cases were diagnosed using echocardiography and confirmed by surgery. One of the main exposures of interest was maternal dietary habits in early pregnancy. Based on prior food frequency surveys and local eating customs, a self-administered food frequency questionnaire was developed (23, 24). When children were taken to the hospital for tests or surgery, professionally trained investigators conducted a face-to-face interview with their mothers to fill out this questionnaire reflecting the maternal dietary habits throughout the early pregnancy.

The questionnaire has established content validity, test–retest reliability (*r* = 0.867), and internal consistency (α = 0.811). It focuses on maternal dietary habits in terms of pickled vegetables, smoked foods, grilled foods, fried foods, fresh meat, fish, and shrimp, fresh eggs, fresh vegetables or fruits, soy foods, and milk products. In this study, the frequency of food consumption was defined as follows: (1) hardly intake was defined as less than or equal to two times per week; (2) sometimes intake was defined as three to five times per week; and (3) often intake was defined as more than or equal to six times per week.

In consideration of potential confounders, the questionnaire also included several characteristics as covariates, and the following information was preselected: age at pregnancy onset (< *25, 25–29, 30–34, or* ≥*35*), family income in the past 1 year (< *50,000, 50,000–100,000, 100,000–150,000*, ≥*150,000 RMB*), residence (*rural or urban*), maternal education level (< *9, 9–12, 13–16, or* ≥*17 years*), maternal pre-pregnancy body mass index (BMI) (< *18.5, 18.5–23.9, 24–26.9, or* ≥*27*), active smoking (*do you have a smoking experience in 3 months before pregnancy and/or during the first-trimester pregnancy, yes or no*), drink (*do you have a drinking alcohol experience in 3 months before pregnancy and/or during the first-trimester pregnancy, yes or no*), folic acid supplementation use (*do you use any folic acid supplementation in 3 months before pregnancy and/or during the first-trimester pregnancy, yes or no*), antibiotic use (*do you use any antibiotic (i.e.*, β*-Lactams, aminoglycosides, macrolides, tetracycline, quinolones, and chloramphenicol) in the 3 months before pregnancy and/or during the first-trimester pregnancy, yes or no)*, gestational diabetes (*yes or no*), gestational hypertension (*yes or no*), history of congenital heart diseases in family (*yes or no*), and consanguineous marriage (*yes or no*).

### SNP Selection and Genotyping

When mothers completed the abovementioned questionnaires, they were asked to provide 3 to 5 ml of peripheral venous blood for genotyping. Blood samples were collected in EDTA-treated (ethylenediamine tetraacetic acid) anticoagulant tubes immediately after blood sampling and centrifuged to separate plasma and blood cells. Blood cells were isolated and kept at −80°C until genotyping. The *MTHFD1* gene candidate loci were chosen based on earlier published research that indicated *MTHFD1* gene polymorphisms (22) and their interactions with maternal dietary habits influenced developmental abnormalities such as CHD (16, 21). As a result, rs1950902, rs2236225, and rs2236222 were eventually chosen as candidate loci in our study.

Briefly, SNP markers were selected using the SNPBrowser™ program (version 3.0) provided by AppliedBiosystems Inc. This program allowed selection of SNP markers from the HapMap database (http://www.hapmap.org/). We excluded these SNPs with minor allele frequencies <10% in Caucasians. The polymorphisms of the *MTHFD1* gene were tested using a matrix-assisted laser desorption and ionization time-of-flight mass spectrometry MassARRAY system (Agena iPLEX assay, San Diego, CA, USA). The lab technicians who did the genotyping, retyped and double-checked each sample, and recorded the genotype data were unaware of whether the samples were from cases or controls. We imposed a minimum SNP genotyping call rate at the level of 50%, which was applied to ensure data integrity of the individual's genotypes that had been called. Successful rates for SNPlex assays were all >90% for the three SNPs.

### Statistical Analysis

Statistical analysis was performed using R software, version 3.5.0 (R Foundation for Statistical Computing). All tests were two-tailed, and *P* < .05 was considered to indicate a statistically significant difference except where otherwise specified. To minimize type I error, a false discovery rate *P* value (FDR_*P*) was applied to multiple test corrections based on Benjamini–Hochberg. The statistically significant results should meet FDR_*P* < 0.05 if applicable.

The distribution of the individual's baseline characteristics in the study population was presented as a number (proportion) for categorical data. Whether or not the differences between cases and controls were substantial was tested with chi-square for categorical variables. Hardy–Weinberg equilibrium (HWE) was tested for the control group (significance level at *P* < 0.05). We comprehensively analyzed the association of genotype and three genetic models (i.e., dominant, recessive, and additive models) for every SNP with the risk of VSD. The dominant model meant heterozygote and mutant type homozygote vs. wild type homozygote, the recessive model meant mutant type homozygote vs. heterozygote and wild type homozygote, the additive model meant mutant type homozygote vs. heterozygote vs. mutant type homozygote.

Odds ratios (OR) and their 95% confidence intervals (CIs) were used to show the strength of association. The main effect of maternal dietary habits and *MTHFD1* gene polymorphisms was estimated by aOR in multivariate logistic regression models to control for potential confounders. Furthermore, the interaction effect of *MTHFD1* gene polymorphisms and maternal dietary habits was also examined by logistic regression models.

## Results

### Characteristics of Study Participants

In this study, we recruited 864 eligible participants, including 360 mothers of infants with VSD in the case group and 504 mothers of children without a congenital malformation in the control group. Among 360 children with VSD, 50 (13.9 %) had atrial septal defect, 98 (27.2 %) had patent ductus arteriosus, 6 (1.7 %) had aorto-pulmonary window, 2 (0.6 %) had complete transposition of great arteries, and 24 (6.7 %) had tetralogy of Fallot, but 206 (57.2%) had no other subtypes of CHD. Considering that some cases were diagnosed with multiple subtypes of CHD, the sum of the various subtypes was not equal to 360. Comparisons of baseline characteristics across the two groups are summarized in Table 1. Our study shows that there are statistically significant differences between groups for the following characteristics: age at pregnancy onset, residence, maternal education level, active smoking, drink, folic acid use, gestational diabetes, gestational hypertension, history of congenital heart diseases in family, and consanguineous marriage (all *P* values <0.05). However, we did not observe statistically significant differences in family income in the past 1 year (*P* = 0.100), antibiotic use (*P* = 0.092), and pre-pregnancy BMI (*P* = 0.108) between the case and control groups. As a result, when evaluating the associations of maternal dietary habits, SNPs of *MTHFD1* gene polymorphisms, and their interactions with the risk of VSD in offspring, these statistically significant factors were adjusted as confounders.

**Table 1:** Baseline characteristics in case and control groups^a^.

**Characteristics**	**Control group** **(***n*** = 504)**	**Case group** **(***n*** = 360)**	**Univariable analysis^b^**
Age at pregnancy onset			*χ^2^* = 7.976; *P* = 0.047
<25	98 (19.4%)	98 (27.2%)	
25–29	210 (41.7%)	144 (40.0%)	
30–34	126 (25.0%)	74 (20.6%)	
≥35	70 (13.9%)	44 (12.2%)	
Family income in the past 1 year (RMB)			*χ^2^* = 6.249; *P* = 0.100
<50,000	144 (28.6%)	127 (35.3%)	
50,000–100,000	216 (42.9%)	142 (39.4%)	
10,000–1,50,000	46 (9.1%)	37 (10.3%)	
≥1,50,000	98 (19.4%)	54 (15.0%)	
Residence			*χ^2^* = 29.011; *P* = 0.000
Rural	276 (54.8%)	262 (72.8%)	
Urban	228 (45.2%)	98 (27.2%)	
Maternal education level (years)			*χ^2^* = 121.125; *P* = 0.000
<9	6 (1.2%)	38 (10.6%)	
9–12	100 (19.8%)	152 (42.2%)	
13–16	168 (33.3%)	106 (29.4%)	
≥17	230 (45.6%)	64 (17.8%)	
Pre-pregnancy BMI^c^			*χ^2^* = 6.068; *P* = 0.108
<18.5	138 (27.4%)	74 (20.6%)	
18.5–23.9	282 (56.0%)	222 (61.70%)	
24–26.9	52 (10.3%)	44 (12.2%)	
≥27	32 (6.3%)	20 (5.6%)	
Active smoking			*χ^2^* = 10.029; *P* = 0.003
No	494 (98.0%)	338 (93.9%)	
Yes	10 (2.0%)	22 (6.1%)	
Drink			*χ^2^* = 7.763; *P* = 0.006
No	468 (92.9%)	314 (87.2%)	
Yes	36 (7.1%)	46 (12.8%)	
Folic acid use			*χ^2^* = 12.207; *P* = 0.000
No	34 (6.7%)	50 (13.9%)	
Yes	470 (93.3%)	310 (86.1%)	
Antibiotic use			*χ^2^* = 2.831; *P* = 0.092
No	496 (98.4%)	348 (96.7%)	
Yes	8 (1.6%)	12 (3.3%)	
Gestational diabetes			*χ^2^* = 12.453; *P* = 0.000
No	482 (95.6%)	322 (89.4%)	
Yes	22 (4.4%)	38 (10.6%)	
Gestational hypertension			χ^2^ = 5.800; *P* = 0.021
No	488 (96.8%)	336 (93.3%)	
Yes	16 (3.2%)	24 (6.7%)	
History of congenital heart diseases in family			*χ^2^* = 12.378; *P* = 0.001
No	500 (99.2%)	344 (95.6%)	
Yes	4 (0.8%)	16 (4.4%)	
Consanguineous marriage			*χ^2^* = 14.089; *P* = 0.000
No	502 (99.6%)	346 (96.1%)	
Yes	2 (0.4%)	14 (3.9%)	

*BMI, body mass index*.

a *Data presented as number (percentage) unless otherwise indicated*.

b *P < 0.05 was considered to indicate a statistically significant difference*.

c *Classification according to Chinese standard for obesity BMI*.

### Maternal Dietary Habits and the Risk of VSD in Offspring

Table 2 shows the frequency of maternal dietary intake throughout early pregnancy. Pickled vegetables, smoked foods, grilled foods, fried foods, fresh meat, fish and shrimp, fresh eggs, fresh fruits or vegetables, soy foods, and milk products were observed to be significantly associated with the risk of VSD in offspring (all *P* values <0.05). To control for potential confounders between the two groups, we used multivariate logistic regression to assess the associations of maternal dietary habits with the risk of VSD (Table 2). Maternal excessive intake of pickled vegetables (aOR = 1.85, 95%CI: 1.45–2.37), smoked foods (aOR = 1.93, 95%CI: 1.48–2.51), barbecued foods (aOR = 1.74, 95%CI: 1.28–2.36), and fried foods (aOR = 1.68, 95%CI: 1.30–2.17) were shown to be associated with a higher risk of VSD in offspring. In contrast, we observed that maternal excessive intake of fresh meat (aOR = 0.61, 95%CI: 0.47–0.79), fish and shrimp (aOR = 0.29, 95%CI: 0.23–0.38), fresh eggs (aOR = 0.54, 95%CI: 0.42–0.70), fresh fruits or vegetables (aOR = 0.44, 95%CI: 0.33–0.60), soy foods (aOR = 0.65, 95%CI: 0.53–0.80), and milk products (aOR = 0.49, 95%CI: 0.40–0.59) could significantly contribute to a lower risk of VSD in offspring.

**Table 2:** Maternal dietary habits and the risk of VSD in offspring^a^.

**Maternal dietary habits**	**Control group** **(***n*** = 504)**	**Case group** **(***n*** = 360)**	**Univariate analysis**	**Unadjusted OR (95% CI)**	**Adjusted OR (95% CI)^b^**
Pickled vegetables			*χ^2^* = 18.382; *P* = 0.000	1.60 (1.29–1.98)	1.85 (1.45–2.37)
Hardly	330 (65.5%)	188 (52.2%)		1	1
Sometimes	148 (29.4%)	134 (37.2%)		1.59 (1.18–2.13)	1.87 (1.34–2.61)
Often	26 (5.2%)	38 (10.6%)		2.57 (1.51–4.36)	3.40 (1.87–6.17)
Smoked foods			*χ^2^* = 20.562; *P* = 0.000	1.69 (1.34–2.13)	1.93 (1.48–2.51)
Hardly	276 (54.8%)	149 (41.4%)		1	1
Sometimes	212 (42.1%)	182 (50.6%)		1.59 (1.20–2.11)	1.91 (1.38–2.63)
Often	16 (3.2%)	29 (8.1%)		3.36 (1.77–6.38)	3.82 (1.87–7.81)
Barbecued foods			*χ^2^* = 18.455; *P* = 0.000	1.77 (1.35–2.31)	1.74 (1.28–2.36)
Hardly	376 (74.6%)	225 (62.5%)		1	1
Sometimes	124 (24.6%)	123 (34.2%)		1.66 (1.23–2.24)	1.70 (1.21–2.38)
Often	4 (0.8%)	12 (3.3%)		5.01 (1.6–15.73)	3.66 (1.06–12.57)
Fried foods			*χ^2^* = 15.235; *P* = 0.000	1.53 (1.23–1.91)	1.68 (1.30–2.17)
Hardly	306 (60.7%)	180 (50%)		1	1
Sometimes	178 (35.3%)	147 (40.8%)		1.40 (1.06–1.87)	1.82 (1.31–2.53)
Often	20 (4%)	33 (9.2%)		2.81 (1.56–5.04)	2.40 (1.25–4.58)
Fresh meat			*χ^2^* = 28.493; *P* = 0.000	0.55 (0.43–0.69)	0.61 (0.47–0.79)
Hardly	22 (4.4%)	53 (14.7%)		1	1
Sometimes	20 (4%)	14 (3.9%)		0.29 (0.13–0.68)	0.27 (0.10–0.68)
Often	462 (91.7%)	293 (81.4%)		0.26 (0.16–0.44)	0.32 (0.18–0.56)
Fish and shrimp			*χ^2^* = 154.281; *P* = 0.000	0.25 (0.20–0.32)	0.29 (0.23–0.38)
Hardly	22 (4.4%)	80 (22.2%)		1	1
Sometimes	146 (29%)	186 (51.7%)		0.35 (0.21–0.59)	0.38 (0.22–0.66)
Often	336 (66.7%)	94 (26.1%)		0.08 (0.05–0.13)	0.10 (0.06–0.18)
Fresh eggs			*χ^2^* = 58.659; *P* = 0.000	0.44 (0.35–0.56)	0.54 (0.42–0.70)
Hardly	24 (4.8%)	44 (12.2%)		1	1
Sometimes	56 (11.1%)	96 (26.7%)		0.94 (0.52–1.70)	1.07 (0.55–2.07)
Often	424 (84.1%)	220 (61.1%)		0.28 (0.17–0.48)	0.41 (0.23–0.73)
Fresh fruits or vegetables			*χ^2^* = 70.061; *P* = 0.000	0.38 (0.29–0.50)	0.44 (0.33–0.60)
Hardly	10 (2%)	66 (18.3%)		1	1
Sometimes	26 (5.2%)	14 (3.9%)		0.08 (0.03–0.21)	0.07 (0.03–0.19)
Often	468 (92.9%)	280 (77.8%)		0.09 (0.05–0.18)	0.12 (0.06–0.24)
Soy foods			*χ^2^* = 53.235; *P* = 0.000	0.52 (0.43–0.62)	0.65 (0.53–0.80)
Hardly	70 (13.9%)	107 (29.7%)		1	1
Sometimes	146 (29%)	131 (36.4%)		0.59 (0.40–0.86)	0.72 (0.47–1.10)
Often	288 (57.1%)	122 (33.9%)		0.28 (0.19–0.40)	0.43 (0.28–0.65)
Milk products			*χ^2^* = 125.216; *P* = 0.000	0.43 (0.36–0.50)	0.49 (0.40–0.59)
Hardly	82 (16.3%)	187 (51.9%)		1	1
Sometimes	120 (23.8%)	55 (15.3%)		0.20 (0.13–0.30)	0.23 (0.15–0.37)
Often	302 (59.9%)	118 (32.8%)		0.17 (0.12–0.24)	0.23 (0.15–0.33)

*VSD, ventricular septal defect; OR, odds ratio; CI, confidence interval*.

a *Data presented as number (percentage) unless otherwise indicated*.

b *Adjusted for age at pregnancy onset, residence, education level, active smoking, drink, folic acid use, gestational diabetes, gestational hypertension, history of congenital heart diseases in family and consanguineous marriage*.

### Maternal *MTHFD1* Gene Polymorphisms and the Risk of VSD in Offspring

The genotype frequencies of the *MTHFD1* gene at rs1950902, rs2236222, and rs2236225 were all within HWE (all *P* values > 0.05), according to the results of HWE tests in the control group (Table 3).

**Table 3:** Genotypes Frequencies of genetic loci of *MTHFD1* gene and Hardy-Weinberg Disequilibrium tests of the control group^a^.

**Genetic loci**	**Chromosome**	**Major allele**	**Minor allele**	**MAF**	**Group**	**Genotype Frequencies**			***P*** ^b^
						**AA**	**AB**	**BB^c^**	
rs1950902	14:64415662	G	A	0.3829	Control	190 (37.7%)	242 (48.0%)	72 (14.3%)	0.151
					Case	174 (48.3%)	144 (40.0%)	42 (11.7%)	
rs2236225	14:64442127	G	A	0.1910	Control	332 (65.9%)	152 (30.2%)	20 (4.0%)	0.620
					Case	226 (62.8%)	118 (32.8%)	16 (4.4%)	
rs2236222	14:64448464	A	G	0.2065	Control	318 (63.1%)	164 (32.5%)	22 (4.4%)	0.883
					Case	198 (55.0%)	126 (35.0%)	36 (10.0%)	

*MTHFD1, Methylenetetrahydrofolate dehydrogenase 1; MAF, minimum allele frequency*.

a *Data presented as number (percentage) unless otherwise indicated*.

b *P < 0.05 was considered to indicate a statistically significant difference*.

c *AA, Homozygous with minor allele*.

*AB, Heterozygous*.

*BB, Homezygous with major allele*.

According to the univariate analyses (Table 4), the genetic polymorphisms of *MTHFD1* gene at rs1950902 (the dominant model: FDR_*P* = 0.008) and rs2236222 (the dominant model: FDR_*P* = 0.036) between the case and control groups were statistically significant. Taking into account potential confounders, we then applied multivariate logistic regression to determine the associations of *MTHFD1* gene polymorphisms on the risk of VSD (Table 4). After adjustment for baseline data, the genetic polymorphisms of the maternal *MTHFD1* gene at rs1950902 were shown to be significantly associated with a lower risk of VSD in offspring (GA *vs*. GG: aOR = 0.67, 95%CI: 0.50–0.90; the dominant model: aOR = 0.66, 95%CI: 0.50–0.88; the additive model: aOR = 0.76, 95%CI: 0.62–0.93). Additionally, the genetic polymorphisms of maternal *MTHFD1* gene at rs2236222 were significantly associated with a higher risk of VSD in offspring (GG *vs*. AA: aOR = 2.75, 95%CI: 1.57–4.83; the dominant model: aOR = 1.44, 95%CI: 1.09–1.90; the recessive model: aOR = 2.52, 95%CI: 1.45–4.38; the additive model: aOR = 1.46, 95%CI: 1.17–1.82). However, we found no statistically significant associations between maternal *MTHFD1* gene polymorphisms at rs2236225 and the risk of VSD.

**Table 4:** Associations between maternal polymorphisms of *MTHFD1* gene and the risk of VSD.

**SNPs**	**Univariate logistic regression**		**Multivariate logistic regression**	
	**Unadjusted OR (95% CI)**	**FDR_** ***P***	**Adjusted OR (95% CI)^b^**	***FDR_P*** ^a^
rs1950902				
G/G	1.00		1.00	
G/A	**0.65 (0.49–0.87)**	**0.012**	**0.67 (0.50–0.90)**	**0.021**
A/A	0.64 (0.41–0.98)	0.077	0.65 (0.42–1.00)	0.098
Dominant model^c^	**0.65 (0.49–0.85)**	**0.008**	**0.66 (0.50–0.88)**	**0.015**
Recessive mode^d^	0.79 (0.53–1.19)	0.395	0.80 (0.53–1.20)	0.417
Additive model^e^	**0.75 (0.61–0.92)**	**0.013**	**0.76 (0.62–0.93)**	**0.021**
rs2236225				
G/G	1.00		1.00	
G/A	1.14 (0.85–1.53)	0.440	1.15 (0.86–1.55)	0.469
A/A	1.18 (0.60–2.32)	0.687	1.01 (0.51–2.03)	0.976
Dominant model	1.14 (0.86–1.52)	0.440	1.14 (0.86–1.51)	0.474
Recessive model	1.13 (0.57–2.20)	0.730	0.97 (0.49–1.92)	0.976
Additive model	1.12 (0.88–1.42)	0.440	1.09 (0.86–1.39)	0.554
rs2236222				
A/A	1.00		1.00	
A/G	1.23 (0.92–1.65)	0.265	1.27 (0.94–1.70)	0.195
G/G	**2.63 (1.50–4.60)**	**0.005**	**2.75 (1.57–4.83)**	**0.000**
Dominant model	**1.40 (1.06–1.84)**	**0.036**	**1.44 (1.09–1.90)**	**0.021**
Recessive model	**2.43 (1.41–4.21)**	**0.005**	**2.52 (1.45–4.38)**	**0.005**
Additive model	**1.43 (1.15–1.78)**	**0.005**	**1.46 (1.17–1.82)**	**0.005**

*VSD, ventricular septal defect; MTHFD1, Methylenetetrahydrofolate dehydrogenase 1; OR, odds ratio; CI, confidence interval; FDR_P, false discovery rate P value*.

a *FDR_P < 0.05 was considered to indicate a statistically significant difference*.

b *Adjusted for age at pregnancy onset, residence, maternal education level, active smoking, drink, folic acid use, gestational diabetes, gestational hypertension, history of congenital heart diseases in family and consanguineous marriage*.

c *The dominant model means heterozygote and mutant type homozygote vs. wild type homozygote*.

d *The recessive model means mutant type homozygote vs. heterozygote and wild type homozygote*.

e *The additive model means mutant type homozygote vs. heterozygote vs. mutant type homozygote*.

### Interaction Effects of Maternal Dietary Habits and the *MTHFD1* Gene on the Risk of VSD in Offspring

Multivariate logistic regression was used to determine if maternal dietary habits and *MTHFD1* gene polymorphisms had statistically significant interaction effects on the development of VSD in children (Table 5). Multivariate logistic regression was applied to determine if maternal dietary habits and *MTHFD1* gene polymorphisms had statistically significant interaction effects on the development of VSD in offspring (Table 5). For rs1950902, significant interactions of the variant genotypes (GA+AA) and excessive intake of fresh meat (aOR = 0.84, 95%CI: 0.75–0.93), fish and shrimp (aOR = 0.76, 95%CI: 0.68–0.86), fresh eggs (aOR = 0.85, 95%CI: 0.76–0.95), fruits or vegetables (aOR = 0.82, 95%CI: 0.74–0.91), soy foods (aOR = 0.82, 95%CI: 0.72–0.93), and milk products (aOR = 0.77, 95%CI: 0.68–0.87) were observed.

**Table 5:** Interactions of maternal polymorphisms of *MTHFD1* gene and maternal dietary habits by multivariate logistic regression.

**Dietary habits^a^**	**Interaction with rs1950902^b^**		**Interaction with rs2236225^b^**		**Interaction with rs2236222^b^**	
	**aOR (95%CI)^d^**	***FDR_P*** ^c^	**aOR (95%CI)^d^**	***FDR_P*** ^c^	**aOR (95%CI)^d^**	***FDR_P*** ^c^
Pickled vegetables	0.96 (0.81–1.15)	0.686	1.34 (1.11–1.61)	**0.020**	1.44 (1.19–1.74)	**0.000**
Smoked foods	0.95 (0.80–1.14)	0.653	1.18 (0.98–1.42)	0.208	1.37 (1.14–1.63)	**0.005**
Barbecued foods	0.89 (0.72–1.09)	0.355	1.28 (1.04–1.58)	0.073	1.39 (1.12–1.72)	**0.010**
Fried foods	0.91 (0.76–1.08)	0.355	1.32 (1.09–1.59)	**0.025**	1.27 (1.06–1.53)	**0.025**
Fresh meat	0.84 (0.75–0.93)	**0.002**	1.02 (0.91–1.14)	0.923	1.08 (0.97–1.21)	0.238
Fish and shrimp	0.76 (0.68–0.86)	**0.000**	0.93 (0.82–1.06)	0.550	1.00 (0.88–1.14)	0.967
Fresh eggs	0.85 (0.76–0.95)	**0.007**	0.99 (0.88–1.11)	0.923	1.10 (0.98–1.23)	0.198
Fruits or vegetables	0.82 (0.74–0.91)	**0.000**	1.01 (0.90–1.12)	0.923	1.07 (0.96–1.19)	0.305
Soy foods	0.82 (0.72–0.93)	**0.002**	1.01 (0.88–1.15)	0.923	1.08 (0.95–1.23)	0.305
Milk products	0.77 (0.68–0.87)	**0.000**	0.94 (0.83–1.08)	0.635	0.99 (0.87–1.13)	0.967

*MTHFD1, Methylenetetrahydrofolate dehydrogenase 1; aOR, adjusted odds ratio; CI, confidence interval; FDR_P, false discovery rate P value*.

a *Maternal dietary habits were classified as hardly and sometimes/often*.

b *Single nucleotide polymorphisms were classified as wild type and variant genotype*.

c *FDR_P < 0.05 was considered to indicate a statistically significant difference*.

d *Adjusted for age at pregnancy onset, residence, education level, active smoking, drink, folic acid use, gestational diabetes, gestational hypertension, history of congenital heart diseases in family and consanguineous marriage*.

With regard to rs2236225, the interactions of the variant genotypes (GA+AA) with excessive intake of pickled vegetables (aOR = 1.34, 95%CI: 1.11–1.61) and fried foods (aOR = 1.32, 95%CI: 1.09–1.59) were shown to be statistically significant.

For rs2236222, we found significant interactions between the variant genotypes (GA + GG) and excessive intake of pickled vegetables (aOR = 1.44, 95%CI: 1.19–1.74), smoked foods (aOR = 1.37, 95%CI: 1.14–1.63), barbecued foods (aOR = 1.39, 95%CI: 1.12–1.72), and fried foods (aOR = 1.27, 95%CI: 1.06–1.53).

## Discussion

Previous studies concentrate on the involvement of particular nutrients in the etiology of CHD (18, 19). In fact, when considering the interactions in distinct nutrients, maternal dietary habits were more appropriate for assessing associations between dietary factors and CHD than a specific nutrient or hazardous substance separately. Based on a case-control study, we, therefore, focused on the associations between maternal dietary habits and the risk of VSD in offspring. Our findings suggest that mothers who adhered to excessive intakes of pickled vegetables (aOR = 1.85), smoked foods (aOR = 1.93), barbecued foods (aOR = 1.74), and fried foods (aOR = 1.68) during their early pregnancy were more likely to have a VSD-affected infant.

Pickled vegetables contain relatively large amounts of nitrite and N-nitroso compounds. Approximately 85% of dietary nitrate was derived from vegetables with the remaining 15% derived primarily from drinking water (25). It was demonstrated that maternally consumed nitrate/nitrite was capable of permeating the placenta and affecting the developing fetus. Also, previous epidemiologic studies show the positive associations between maternal exposure to nitrate, nitrite, and N-nitroso compounds from diet or drinking water and an increased risk of congenital abnormalities, such as neural tube defects, oral cleft malformations, limb deficiencies, and CHD (25, 26), which provided evidence for our findings. Additionally, smoked, barbecued, and fried foods were a common dietary source of polycyclic aromatic hydrocarbons (PAHs). Because of their capacity to easily cross cellular membranes, including the embryonic and fetal blood–brain barrier, PAHs were considered teratogens, resulting in increased oxidative stress and DNA damage (27). A variety of birth outcomes and anomalies are reported as a result of PAH exposure, and evidence suggests that maternal exposure to PAHs during early pregnancy is linked to CHD (28), which may partially explain our observation of a positive association between maternal excessive intakes of smoked, barbecued, and fried foods and an increased VSD risk in offspring.

In this study, we also found that maternal excessive consumption of fish and shrimp (aOR = 0.29), fresh fruits or vegetables (aOR = 0.44), milk products (aOR = 0.49), fresh meat (aOR = 0.61), and soy foods (aOR = 0.65) during early pregnancy was significantly associated with a reduced risk of VSD in offspring. Fish and shrimp, fresh fruits or vegetables, soy foods, and milk products are all common food types that are rich in proteins, lipids, vitamins, and minerals and provide many essential amino acids and fatty acids for growth and maturation. Of note, among all common food types, fish and shrimp have the strongest preventive benefits against the development of VSD. According to one study conducted by Obermann-Borst (21), a high intake of fish and seafood, which belong to the one-carbon-rich dietary pattern, supplied total protein; vitamins B1, B2, B3, B6, and B12; zinc; EPA; and DHA, which was related to a lower risk of CHD in offspring. Zinc serves as a cofactor for -glutamylhydrolase, a folate absorption enzyme, and methionine synthase, a homocysteine-to-methionine conversion enzyme (29). A moderate amount of intake of zinc is proved to be linked with a reduced risk of CHD in the offspring (30). Also, high levels of omega-3 fatty acid (EPA and DHA) intake from fish have a homocysteine-lowering effect, which may explain some of the beneficial effects of fish intake on pregnancy outcome (31).

To date, the role of *MTHFR* gene polymorphisms in birth defects has received a great attention, particularly the C667T variant. However, other essential genes in the folate metabolism pathway, such as the *MTHFD1* gene, also play a crucial role in maintaining the folate-homocysteine balance and should not be overlooked. Experimental evidence suggests that the *MTHFD1* gene can influence embryonic development by altering folate metabolism. Disruption of the *Mthfd1* gene in homozygous mice resulted in early embryonic lethality, whereas mice heterozygous for the disruption appeared healthy but had impaired folate-mediated one-carbon metabolism (32). It is assumed that maternal *MTHFD1* gene polymorphisms are associated with the risk of VSD in offspring. To provide more epidemiological data, we assessed their associations in Chinese populations.

This study concentrated on three SNPs of the *MTHFD1* gene, including two functional non-synonymous polymorphisms with known biochemical phenotypes, rs1950902 and rs2236225, and one synonymous polymorphism, rs2236222. The rs2236225 polymorphism is the well-studied genetic variant of the *MTHFD1* gene, and this SNP has been a hot spot for research into genetic risk factors for birth defects. At nucleotide 1958, *MTHFD1* rs2236225 (G1958A; R653Q) undergoes a G-to-A change, resulting in an arginine-to-glutamate substitution at amino acid 653. The rs1950902 polymorphism (C401T; R134K), which includes a C-to-T transition at nucleotide 401 and results in an arginine to lysine change at amino acid 134, is another well-studied genetic variation of the *MTHFD1* gene. However, among epidemiological studies, the association between *MTHFD1* gene polymorphisms and the risk of VSD has yielded conflicting results. Our findings, as well as Karen (14) and Huang's results (33), suggest that maternal genetic polymorphisms of *MTHFD1* gene at rs2236225 could not affect the susceptibility to CHD in offspring. With respect to the subtypes of CHD, positive associations of *MTHFD1* rs2236225 with atrial septal defects (34), tetralogy of Fallot (35), and aortic stenosis (14) were observed. When it came to the polymorphism rs1950902, there were no epidemiologic studies concerning the association between rs1950902 and VSD but one study involving tetralogy of Fallot. However, in one study by Xu, no evidence for the association between *MTHFD1* 1950902 and tetralogy of Fallot was found (33). These observations need to be replicated, and further investigations are warranted.

In addition, we found a significant interaction between the maternal *MTHFD1* gene and dietary habits on the risk of VSD. This finding seems to be biologically plausible. Previous research has discovered strong connections between dietary habits and folate levels (36). Mothers who consumed comparatively more fish and seafood had higher serum and red blood cell folate levels as compared with those who consumed less fish and seafood (21). Furthermore, Christensen et al. (22) found that low dietary folate interacted with MTHFD1 synthetase deficiency in mice, a model for the R653Q mutation, to greatly increase the risk of birth defects. Nonetheless, the specific mechanism remained unclear, necessitating more research.

The limitations of the study needed to be addressed. First, given that this was hospital-based case-control research, the case and control groups from various clinics in one hospital did not appear to agree on the majority of characteristics. However, we adjusted the baseline characteristics when exploring the associations of maternal dietary habits, *MTHFD1* gene polymorphisms, and their interaction with the risk of VSD in offspring. Second, in consideration of population stratification bias in epidemiologic studies, we recruited the participants restricted to the Han Chinese ethnicity. Due to the obvious ethnic and regional differences in gene polymorphisms, it was necessary to conduct this study in larger and different ethnic populations. Third, because maternal dietary habits during the period of gestation were retrospectively reported by the mother, recall bias was unavoidable. However, to minimize the recall bias, this study only recruited infants who were less one year old in both the case and control groups. One study revealed dietary habits of pregnant women were able to be well-recalled after birth (37). Fourth, this study is mainly concerned with the influence of maternal *MTHFD1* gene polymorphisms on the risk of VSD, whereas the role of the polymorphisms of children was ignored. Further research into the associations between *MTHFD1* gene polymorphisms in children and VSD risk is required, which might provide additional crucial insights into the etiology of VSD. Last but not least, due to the limitation of the lack of replication studies, it is still to be further studied whether our findings can be extended to different populations.

## Conclusion

In this case-control study based on Han Chinese populations, maternal *MTHFD1* rs1950902 and rs2236222 were found to be associated with the risk of VSD, but there was no statistically significant association between maternal *MTHFD1* rs2236225 and the risk of VSD in offspring. Furthermore, maternal dietary habits as well as their interactions with maternal *MTHFD1* gene polymorphisms, have a significant impact in the development of VSD. Due to several limitations in this research, future studies with a larger sample size, a prospective methodology, and different ethnicities are required to corroborate our findings.

## Data Availability Statement

The raw data supporting the conclusions of this article will be made available by the authors, without undue reservation.

## Ethics Statement

This study was performed in line with the principles of the Declaration of Helsinki. Approval was granted by the Ethics Committee of Xiangya School of Public Health Central South University (No. XYGW-2018-36). The patients/participants provided their written informed consent to participate in this study.

## Author Contributions

JD, JL, and YLi performed the experiments. SZ and TW analyzed the data and statistical analyses. JS, YLiu, and MS contributed reagents, material, and analysis tools. XS, PZ, and JQ wrote the main manuscript text. LC, JW, and MS collected reference and managed data. All authors contributed to the article and approved the submitted version.

## Funding

This study was supported by the Project Funded by National Natural Science Foundation Program of China (82073653 and 81803313), Hunan Provincial Key Research and Development Program (2018SK2063 and 2018SK2062), Hunan Provincial Science and Technology Talent Support Project (2020TJ-N07), Natural Science Foundation of Hunan Province (2018JJ2551), Open Project from NHC Key Laboratory of Birth Defect for Research and Prevention (KF2020006), and the 68th General Program Supported by China Postdoctoral Science Foundation (2020M682644).

## Conflict of Interest

The authors declare that the research was conducted in the absence of any commercial or financial relationships that could be construed as a potential conflict of interest.

## Publisher's Note

All claims expressed in this article are solely those of the authors and do not necessarily represent those of their affiliated organizations, or those of the publisher, the editors and the reviewers. Any product that may be evaluated in this article, or claim that may be made by its manufacturer, is not guaranteed or endorsed by the publisher.
